# Artificial intelligence algorithm for predicting mortality of patients with acute heart failure

**DOI:** 10.1371/journal.pone.0219302

**Published:** 2019-07-08

**Authors:** Joon-myoung Kwon, Kyung-Hee Kim, Ki-Hyun Jeon, Sang Eun Lee, Hae-Young Lee, Hyun-Jai Cho, Jin Oh Choi, Eun-Seok Jeon, Min-Seok Kim, Jae-Joong Kim, Kyung-Kuk Hwang, Shung Chull Chae, Sang Hong Baek, Seok-Min Kang, Dong-Ju Choi, Byung-Su Yoo, Kye Hun Kim, Hyun-Young Park, Myeong-Chan Cho, Byung-Hee Oh

**Affiliations:** 1 Artificial Intelligence and Big Data Center, Sejong Medical Research Institute, Gyunggi, Korea; 2 Department of Emergency Medicine, Mediplex Sejong Hospital, Incheon, Korea; 3 Division of Cardiology, Cardiovascular Center, Mediplex Sejong Hospital, Incheon, Korea; 4 Department of Internal Medicine, Asan Medical Center, University of Ulsan College of Medicine, Seoul, Korea; 5 Department of Internal Medicine, Seoul National University Hospital, Seoul, Korea; 6 Department of Internal Medicine, Sungkyunkwan University College of Medicine, Seoul, Korea; 7 Department of Internal Medicine, Chungbuk National University College of Medicine, Cheongju, Korea; 8 Department of Internal Medicine, Kyungpook National University College of Medicine, Daegu, Korea; 9 Department of Internal Medicine, College of Medicine, The Catholic University of Korea, Seoul, Korea; 10 Department of Internal Medicine, Yonsei University College of Medicine, Seoul, Korea; 11 Department of Internal Medicine, Seoul National University Bundang Hospital, Seongnam, Korea; 12 Department of Internal Medicine, Yonsei University Wonju College of Medicine, Wonju, Korea; 13 Department of Internal Medicine, Heart Research Center of Chonnam National University, Gwangju, Korea; 14 Division of Cardiovascular and Rare Diseases, Korea National Institute of Health, Cheongju, Korea; Universita degli Studi di Napoli Federico II, ITALY

## Abstract

**Aims:**

This study aimed to develop and validate deep-learning-based artificial intelligence algorithm for predicting mortality of AHF (DAHF).

**Methods and results:**

12,654 dataset from 2165 patients with AHF in two hospitals were used as train data for DAHF development, and 4759 dataset from 4759 patients with AHF in 10 hospitals enrolled to the Korean AHF registry were used as performance test data. The endpoints were in-hospital, 12-month, and 36-month mortality. We compared the DAHF performance with the Get with the Guidelines–Heart Failure (GWTG-HF) score, Meta-Analysis Global Group in Chronic Heart Failure (MAGGIC) score, and other machine-learning models by using the test data. Area under the receiver operating characteristic curve of the DAHF were 0.880 (95% confidence interval, 0.876–0.884) for predicting in-hospital mortality; these results significantly outperformed those of the GWTG-HF (0.728 [0.720–0.737]) and other machine-learning models. For predicting 12- and 36-month endpoints, DAHF (0.782 and 0.813) significantly outperformed MAGGIC score (0.718 and 0.729). During the 36-month follow-up, the high-risk group, defined by the DAHF, had a significantly higher mortality rate than the low-risk group(*p*<0.001).

**Conclusion:**

DAHF predicted the in-hospital and long-term mortality of patients with AHF more accurately than the existing risk scores and other machine-learning models.

## Introduction

Approximately 26 million adults worldwide have heart failure, and acute heart failure (AHF) is the leading cause of hospitalization in Europe and the United States, resulting in more than 1 million admissions, and representing 1%–2% of all hospitalizations.[1,2] In the past decades, the mortality rate of AHF has improved with advances in treatment, but AHF is still a leading cause of mortality worldwide.[1–3] Risk stratification and prognosis prediction are critical in identifying high-risk patients and decision making for the treatment of patients with AHF.

There are several mortality prediction models for heart failure, such as Get with the Guidelines-Heart Failure (GWTG-HF) score, Meta-Analysis Global Group in Chronic Heart Failure (MAGGIC) score.[4,5] However, these prognostic models have limitations for the current daily practice. First, GWTG-HF and MAGGIC are limited in specific situations. GWTG-HF and MAGGIC were developed only for in-hospital and long-term mortality, respectively.[4,5] Second, because the accuracies of these methods are unsatisfactory, these methods cannot be used to decide the treatment of the patient. Third, these models use only limited information that is based on a conventional statistical approach, such as multivariate analysis by the logistic regression model that has a potential limitation of information loss.[6–8]

Recently, artificial intelligence algorithm has achieved a high performance in several medical domains, such as image detection and clinical outcome prediction.[9–11] An advantage of deep-learning is the automatic learning of the feature and relationship from a given data.[12] In this study, we developed and validated a deep-learning-based artificial intelligence algorithm for predicting mortality of patients with acute heart failure (DAHF) by using a large data from 12 hospitals.

## Methods

### Study population

**Fig 1:**
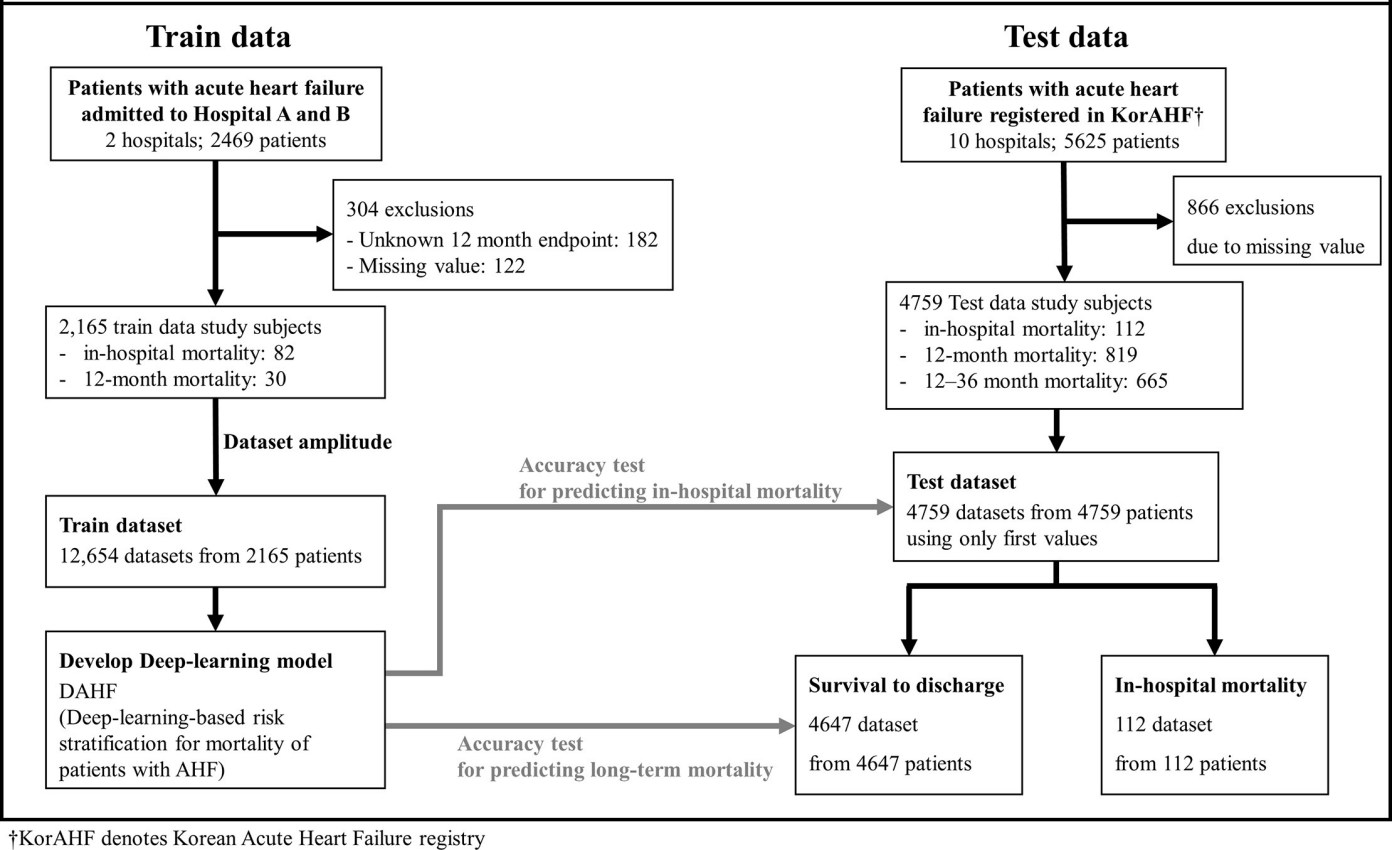
Study flowchart. KorAHF denotes Korean Acute Heart Failure registry.

We conducted a retrospective observational cohort study using AHF patient data from 10 university hospitals of the Korean Acute Heart Failure (KorAHF) registry and 2 hospitals (hospital A: cardiovascular teaching hospital and hospital B: community general hospital), as shown in Fig 1. We defined patients with AHF as patients with signs or symptoms of heart failure and those who met either of the following criteria: 1) lung congestion or 2) objective left ventricular systolic dysfunction or structural heart disease findings.

First, we collected the algorithm train data of patients with AHF admitted in hospitals A and B from October 2016 to December 2017. The data obtained through the electronic health records of the two hospitals were demographic information, treatment and medication, laboratory results, electrocardiography (ECG) and echocardiography findings, final diagnosis, clinical outcome during their hospital stay, and 12-month prognosis after discharge.

Second, we used the data of patients with AHF enrolled in KorAHF as performance test data. KorAHF is a prospective multicenter registry of AHF in Korean patients. All cardiovascular centers in 10 tertiary university hospitals were included in KorAHF from March 2011 to January 2014. The full details of the KorAHF registry’s aims and protocols have been published elsewhere.[13] The data obtained through KorAHF demographic information, treatment and medication, laboratory results, electrocardiography (ECG) and echocardiography findings, final diagnosis, clinical outcome during their hospital stay, 12-month prognosis, and 36-month prognosis.

Because hospitals A and B were not university hospitals, they were excluded in KorAHF. Moreover, the periods of the train data were different with the test data. Therefore, train and test data were completely separated. We excluded patients with missing values of predictor variables and endpoints as shown in Fig 1.

This study was conducted in accordance with the Declaration of Helsinki and the relevant guidelines and regulations. The institutional review boards (IRBs) of Sejong General Hospital (2018–0839) and Mediplex Sejong Hospital (2018–073) approved this study protocol and granted waivers of informed consent based on general impracticability and minimal harm. Patient information was anonymized and de-identified before the analysis. KorAHF data were collected by each site and approved by the IRB at each hospital. The KorAHF committee approved and provided data for the present study.

### Data management

We used the data of hospital A and B as train data for prediction algorithm development. And we used the data of KorAHF as test data to confirm whether the DAHF can be applied to other hospitals after development. These two data were completely separated.

The DAHF is a risk stratification model for predicting in-hospital mortality and long-term mortality of patients with AHF at the time of admission. We used the demographic information, ECG, echocardiography, and laboratory data of patients with AHF, including age (years), sex (male or female), body mass index (BMI, kg/m^2^), systolic blood pressure (SBP, mmHg), diastolic blood pressure (DBP, mmHg), heart rate (HR, bpm), present atrial fibrillation (Afib, yes or no), QRS duration (QRS, ms), corrected QT interval (QTc, ms), left atrial dimension (LAD, mm), left ventricular dimension end-diastole (LVDd, mm), left ventricular dimension end-systole (LVDs, mm), ejection fraction (EF, %), white blood cell (WBC, /mL), hemoglobin (g/dL), platelet (/mL), albumin (g/dL), sodium (mmol/L), potassium (mmol/L), blood urea nitrogen (BUN, mg/dL), creatinine (Cr, mg/dL), and glucose (mg/dL), as the predictor variables.

**Fig 2:**
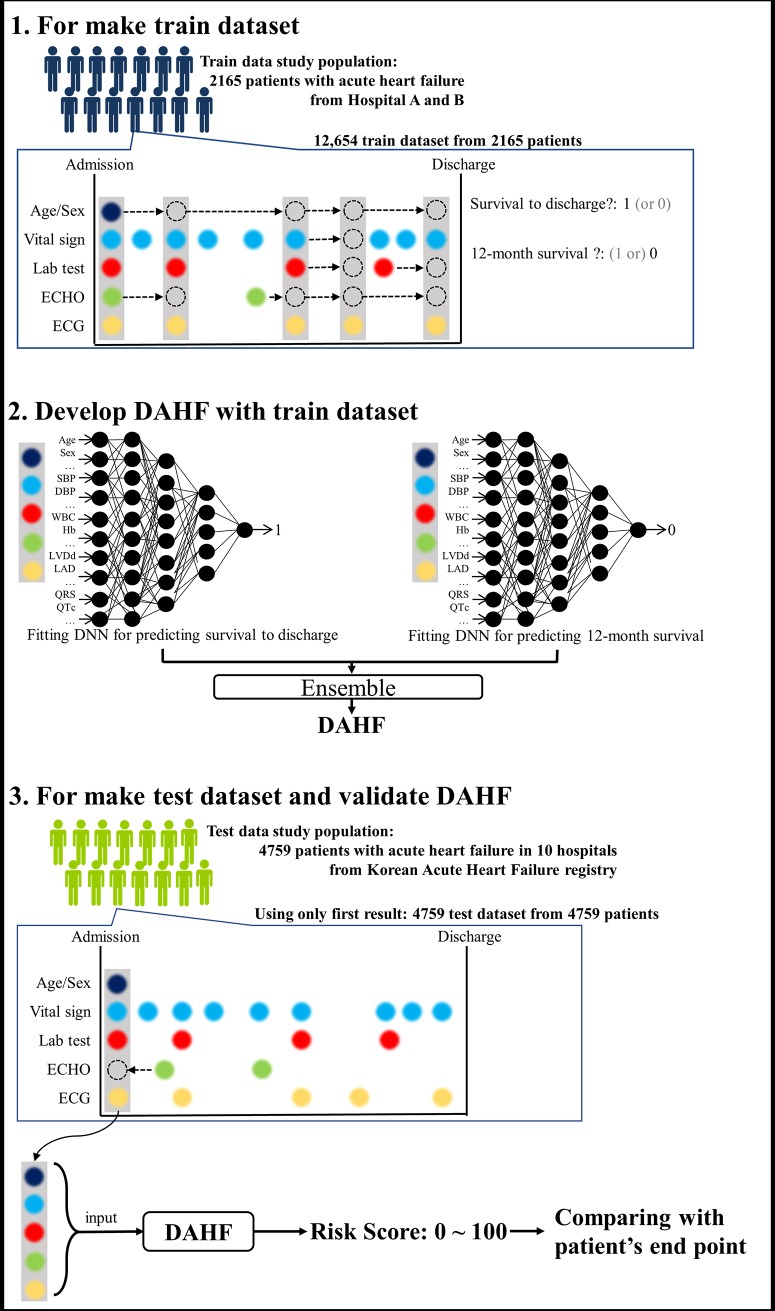
Train and validation of deep-learning prediction model. DAHF denotes deep-learning-based artificial intelligence algorithm for predicting mortality of patients with acute heart failure. Abbreviations: DBP, diastolic blood pressure; DNN, deep neural network; ECHO, echocardiography; ECG, electrocardiography; Hb, hemoglobin; LAD, left atrium dimension; LVDd, left ventricle end-diastolic dimension; QRS, QRS duration; QTc, corrected QT duration.

In the train data of hospital A and B, we made a train dataset each time an ECG was taken during the hospital stay of the patient, as shown in Fig 2. If the value of other variables was missing during ECG, we used the most recent values of the demographic information and vital signs, and echocardiography and laboratory findings, as shown in Fig 2. Hence, several train datasets were generated from one patient. Using this method, we amplified and created a dataset sufficient for developing machine- and deep-learning methods.

In the test data of KorAHF, only initial value of the demographic information, vital sign, ECG, echocardiography, and laboratory data at the time of admission were used. If the value of variable was missing during admission, the first result during hospital stay was used. Because the purpose of test dataset was to assess the accuracy of each prediction model, and the goal of each model was to predict the patient’s prognosis at the time of admission, we generated only one test dataset from one patient.

### Endpoints

The difference of accuracy to predict in-hospital mortality and 12- and 36-month mortality in each prediction model was evaluated. The primary endpoint, in-hospital mortality, was defined as in-hospital death occurring during hospitalization. Readmission within 24 h was considered as the same hospital period. The secondary endpoints, 12- and 36-month mortality, were defined as death within 12 and 36 months, respectively, among patients who survived to discharge, as shown in Fig 1.

### Development of deep- and machine-learning prediction models

We developed the DAHF by using only the train dataset. The DAHF was developed by using deep neural network (DNN), a method of deep-learning with 3 hidden layers, 33 nodes, batch normalization, and dropout layers.[14,15] We used *TensorFlow* (the Google Brain Team, Mountain View, United States) as the backend.[16] In this DNN, we used a rectified linear unit (ReLU) and back propagation as the activation function and method of training, respectively. We developed DAHF by ensembling a DNN model fitted to in-hospital death and a DNN model fitted to 12-month death, as shown in Fig 2. We provided a detailed description, reference, and figure of the DNN as S1 File.

We also developed four machine-learning models, random forest (RF), logistic regression (LR), supportive vector machine (SVM), and Bayesian network (BN), for the performance comparison with DAHF.[17] In the previous studies, these machine-learning models were the most typically used methods and showed better performance than traditional methods in several medical domains.[18,19] We used *randomForest*, *glmulti*, *e1071*, and *bnlearn* packages in R (R Development Core Team, Vienna, Austria) for the development of RF, LR, SVM, and BN models, respectively. Moreover, we also provided the detailed description, references, and figures as S1 File. We analyzed the variable importance of LR, RF, SVM, BN, and DNN by using deviance difference, mean decrease Gini, sensitivity analysis, deviance difference, and AUC difference, respectively.

### Test of prediction model performance

After we developed the DAHF and machine-learning models, we compared the performance of these models with the conventional prediction scoring. We compared the performance for predicting in-hospital mortality with the GWTG-HF scores and compared the performance for predicting 12- and 36-months mortality with the MAGGIC scores.[4,5] We compared the performance of the models by using only the test data not used for the model development. GWTG-HF and MAGGIC scores were well validated and used globally. We used the area under the receiver operating characteristic curve (AUC) as the comparative measure. The AUC is a frequently used metric, and the receiver operating characteristic curve shows the sensitivity against 1-specificity.[20] We evaluated the 95% confidence interval using bootstrapping (10,000 times resampling with replacement).[21] We used *ROCR* package in R (R Development Core Team, Vienna, Austria) for these analyses.

We divided the patients of the test data into the high- and low-risk groups based on the DAHF. In this analysis, we used the data of patients who survived to discharge. The optimal cutoff point of the DAHF score was determined when the Youden J statistics was at maximum.[22] After dividing the patients into risk groups, we estimated the 36-month mortality by risk groups by using the Kaplan–Meier method. We used the *pROC* and *survival* packages in R (R Development Core Team, Vienna, Austria) in this analysis.

## Results

We included 8094 patients with AHF (hospitals A and B: 2469; KorAHF: 5625) in the present study (Fig 1). We excluded 1170 patients because of missing values and endpoints. The study subjects comprised 6724 patients, wherein 194 were in-hospital mortalities. The DNN prediction model, DAHF, was developed using 12,654 train datasets from 2165 patients of hospitals A and B. The performance test was performed using 4759 test datasets from 4759 patients of KorAHF (Fig 2). Table 1 shows the baseline characteristics, and a significant difference was found between the characteristics of the train and test data.

**Table 1:** Baseline characteristics†.

	train data (Hospitals A and B, n = 2165)			Test data (KorAHF, n = 4759)			*p*-value‡
	Survivors (n = 2083)	In-hospital death (n = 82)	*p*-value*	Survivors (n = 4577)	In-hospital death (n = 182)	*p*-value*	0.995
**Demographics**							<0.001
Age (years)	65.1±14.2	74.5±12.3	<0.001	68.2±14.5	70.8±14.5	0.020	<0.001
Male (%)	1246 (59.8)	46 (56.1)	0.576	2411 (52.7)	103 (56.6)	0.336	<0.001
BMI (kg/m^2^)	24.5±11.2	21.9±3.3	0.031	23.4±3.9	22.6±3.6	0.007	<0.001
**Vital signs at admission **							
SBP (mmHg)	120.1±19.9	113.4±22.0	0.003	132.1±30.2	116.8±27.0	<0.001	<0.001
DBP (mmHg)	72.1±13.3	67.7±13.4	0.004	79.2±18.7	70.2±19.3	<0.001	<0.001
HR (bpm)	80.0±21.7	97.6±28.8	<0.001	92.5±25.7	94.6±26.3	0.274	<0.001
**Electrocardiography**							
AF (%)	497 (23.9)	31 (37.8)	0.006	1882 (41.1)	63 (34.6)	0.094	<0.001
QRS duration (ms)	101.9±23.8	107.5±26.3	0.037	106.0±28.2	113.2±30.1	0.001	<0.001
QTc (ms)	462.7±42.0	470.8±43.8	0.087	475.0±46.0	474.7±48.0	0.937	<0.001
**Echocardiography **							
LAD (mm)	44.2±9.6	46.0±14.3	0.095	48.2±9.8	45.9±11.1	0.001	<0.001
LVDd (mm)	51.6±8.1	49.8±9.7	0.056	57.5±10.1	56.6±10.9	0.233	<0.001
LVDs (mm)	36.7±10.3	36.7±11.0	0.962	45.2±12.4	45.6±12.4	0.714	<0.001
EF (%)	44.7±14.0	37.8±14.8	<0.001	37.9±15.5	33.3±15.9	<0.001	<0.001
**Laboratory test**							
WBC (/mL)	7954.7 ±2997.2	13864.1 ±7580.7	<0.001	8494.2 ±3714.4	10639.6 ±5317.2	<0.001	<0.001
Hb (g/dL)	12.5±2.2	10.2±1.9	<0.001	12.5±2.3	11.9±2.3	0.001	0.203
Platelets (/mL)	242,838.69 ±91,419.2	161,573.2 ±101,270.7	<0.001	211,782.8 ±86,736.6	197,033.0 ±146,965.7	0.030	<0.001
Alb(g/dL)	4.0±0.6	2.9±0.7	<0.001	3.7±0.5	3.5±0.5	<0.001	<0.001
Sodium (mmol/L)	138.7±3.6	139.9±7.9	0.006	137.6±4.7	135.1±6.3	<0.001	<0.001
Potassium (mmol/L)	4.2±0.5	4.2±0.7	0.775	4.4±0.7	4.6±0.9	<0.001	<0.001
BUN (mg/dL)	20.7±13.5	38.5±24.2	<0.001	25.6±15.8	36.4±24.8	<0.001	<0.001
Cr (mg/dL)	1.3±1.4	1.8±1.3	0.001	1.4±1.4	1.9±1.7	<0.001	<0.001
Glucose (mg/dL)	130.9±52.8	175.0±94.6	<0.001	153.0±74.8	166.5±83.0	0.018	<0.001

† AF, atrial fibrillation; Alb, albumin; BMI, body mass index; BUN, blood urea nitrogen; Cr, creatinine; DBP, diastolic blood pressure; EF, ejection fraction; Hb, hemoglobin; HR, heart rate; KorAHF, Korean Acute Heart Failure registry; LAD, left atrial dimension; LVDd, left ventricular dimension end-diastole; LVDs, left ventricular dimension end-systole; QTc, corrected QT interval; SBP, systolic blood pressure; WBC, white blood cell.

‡ Alternative hypothesis for this *p*-value: a difference is found between the train and test data groups.

* Alternative hypothesis for this *p*-value: a difference is found between the survivor and in-hospital mortality groups.

**Fig 3:**
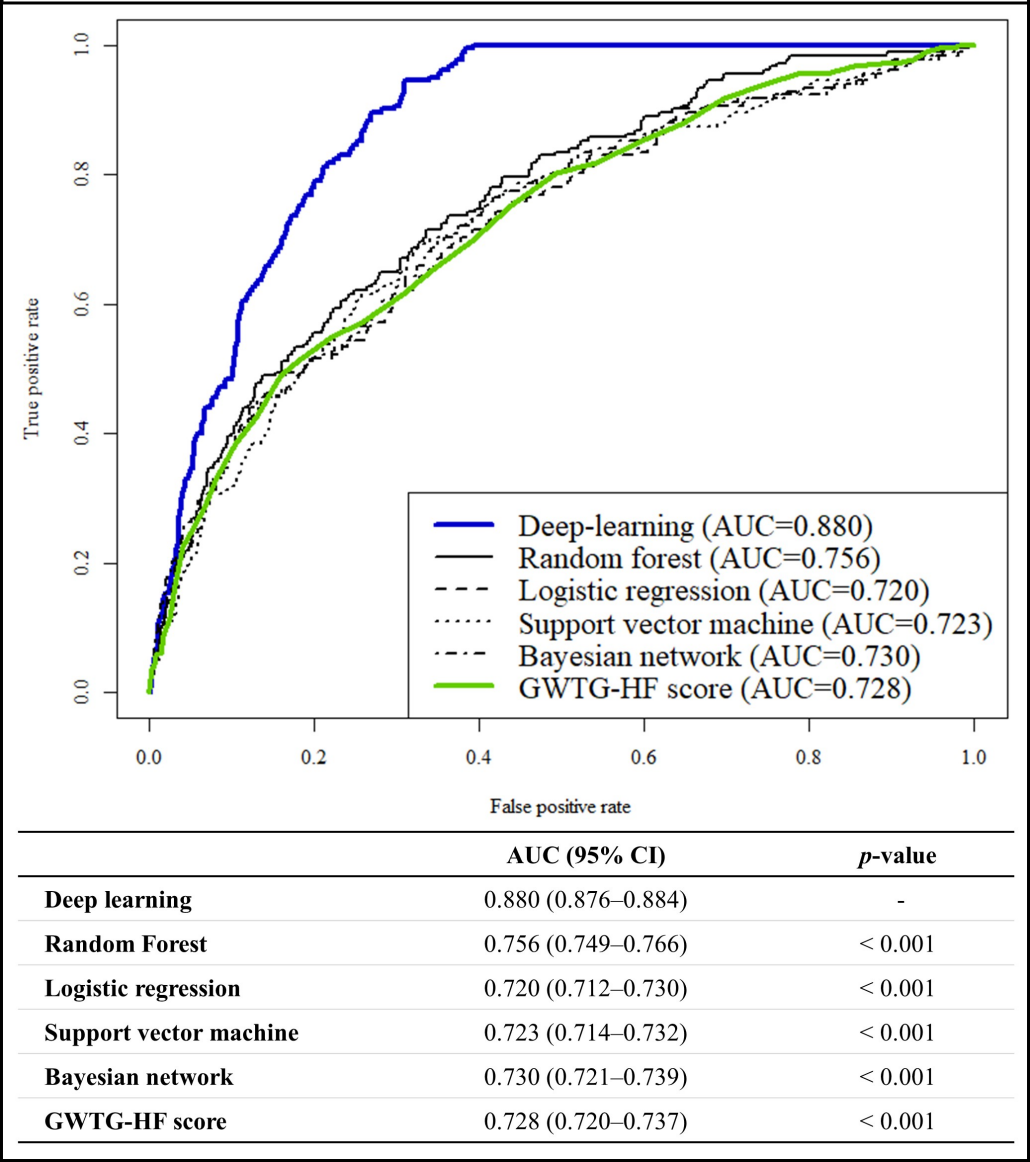
Receiver operating characteristic curve for predicting in-hospital mortality. AUC, area under the receiver operating characteristic curve; CI, confidence interval; GWTG-HF, Get with the Guideline–Heart Failure.

**Fig 4:**
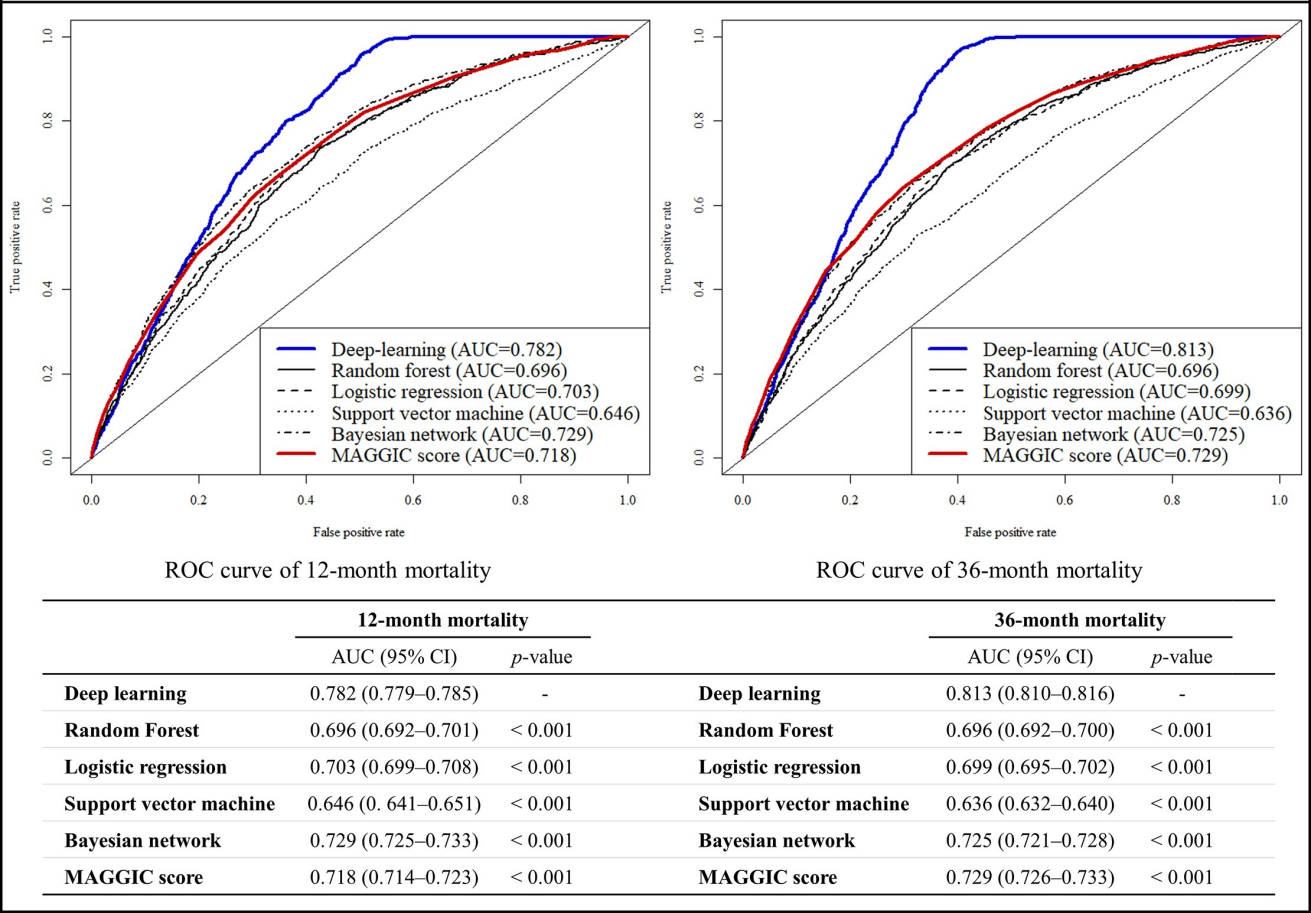
Receiver operating characteristic curves for predicting long-term mortalities. AUC, area under the receiver operating characteristic curve; CI, confidence interval; MAGGIC, Meta-Analysis Global Group in Chronic Heart Failure.

Fig 3 shows that the AUC of the DAHF was 0.880 (95% confidence interval, 0.876–0.884]) during the performance test for predicting in-hospital mortality, and this result significantly outperformed the GWTG-HF score (0.728 [0.720–0.737]) and other machine-learning prediction models. Fig 4 shows that the AUCs of the DAHF were 0.782 [0.779–0.785] and 0.813 [0.810–0.816], respectively, during the performance test for predicting 12- and 36-month mortality. Moreover, these performances significantly outperformed the MAGGIC score (0.718 [0.714–0.723] and 0.729 [0.726–0.733]) and other machine-learning prediction models.

**Fig 5:**
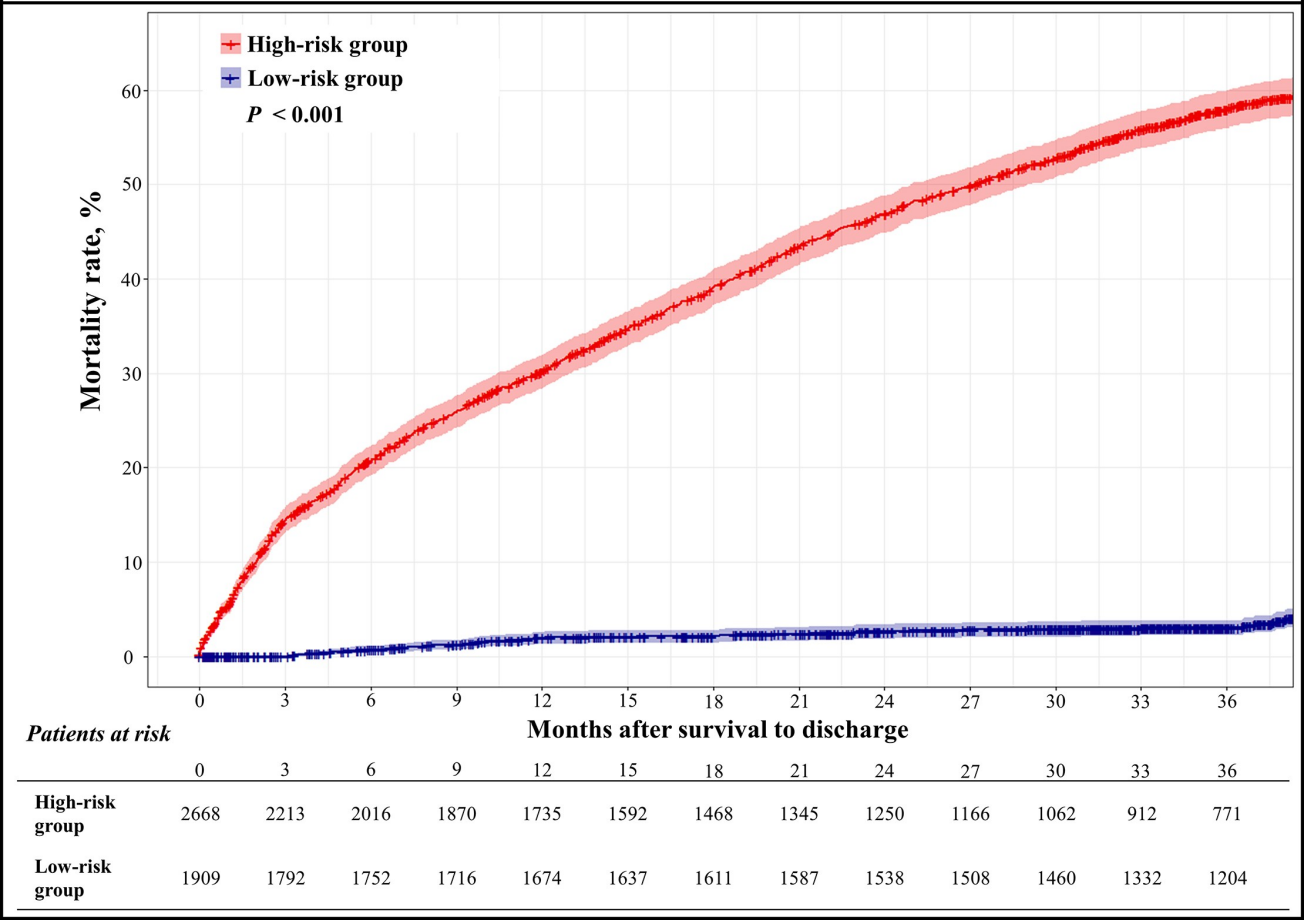
Cumulative hazard of 36-month mortality by deep-learning-based algorithm risk group.

In the train dataset, the optimal cut-off scores of the DAHF risk groups were 0.472. Among 4577 survival-to-discharge patients in the test dataset, the DAHF classified 2668 and 1909 patients as high and low risk, respectively. The cumulative hazard plot of Fig 5. shows that the high-risk group of the DAHF shows a significantly higher hazard than the low-risk group. The high-risk group, defined by the DAHF, has a significantly higher mortality rate than the low-risk group (*p*<0.001).

The characteristics and variable importance of each prediction model is shown in S2 File. The variable importance is different for each prediction model. In the conventional machine-learning models, the *EF* and *QRS duration* variables are less important for prediction. However, for the DAHF, these variables are important for prediction (S2 File). The *BMI* and *LAD* variables are less important for prediction in DAHF than other conventional machine-learning prediction models.

## Discussion

In this study, we developed a deep-learning-based artificial intelligence algorithm, DAHF, for predicting the mortality of patients with AHF using two hospital datasets and validated DAHF using separated AHF registry data. This study revealed that the accuracy of performance of the deep-learning-based artificial intelligence model was excellent for predicting the mortalities and is better than the conventional risk score model. In addition, the deep-learning model outperformed other machine-learning prediction models to predict endpoints. To the best of our knowledge, this study is the first to predict AHF patient endpoints using deep-learning-based artificial intelligence prediction models.

The GWTG-HF and MAGGIC scores are well validated conventional models for risk stratification of AHF patients.[23–26] The previous validation studies have reported that the AUC of the GWTG-HF scores for predicting in-hospital mortality of patients with AHF was 0.71–0.76.[24,25] Furthermore, the AUC of the MAGGIC score for predicting 1–3-year mortality of patients with AHF in previous studies was 0.73–0.74, implying moderate accuracy for predicting the mortality of patients with AHF.[24,26] These results were similar with those of this study.

However, the GWTG-HF and MAGGIC scores have several limitations. First, these models were developed and validated on specific situations. The GWTG-HF was developed to predict in-hospital mortality, and MAGGIC score was developed and validated to predict long-term endpoints.[4,5] Moreover, these prediction scoring methods had no satisfactory performance and were not actively used to decide the treatment of the patient. Because these scoring models were developed by the conventional statistical approach using the logistic regression model that has limitations, including the fixed assumptions on data behavior, and the necessity to preselect variables in the development phase, thus leading to potential information loss. [6–8]

Unlike the conventional statistical approach, deep-learning does not require the preselection of important variables, and the less important variables are naturally ignored in the model fitting process.[12,14,15] Furthermore, deep-learning does not limit the number of input predictive factors and can use all available information without potential loss. Subsequently, the old models cannot reflect the relationship between variables. This is because the risk is measured only by the sum of the variables. Meanwhile, deep-learning obtains the relationship between the variables, as shown in S1 File, unlike conventional methods.[12,14,15]

The previous studies have attempted to predict prognosis, such as readmission and mortalities of AHF, by using conventional machine-learning prediction models.[18,19] In the present study, we confirmed that DNN outperformed other conventional machine-learning prediction models, such as RF, LR, SVM, and BN. The machine-learning prediction model required careful engineering to design a feature extractor for selecting important variables.[17] This process requires a lot of manpower, and loss of important information is possible. Deep-learning, DNN, including feature-learning process, which is a set of methods that allow a model to be fed with raw data and to automatically identify the feature needed for conducting a task.[12,14,15] Deep-learning comprises multiple processing layers of feature as shown in S1 File, obtained by composing simple but non-linear modules, each of which transforms the feature at one level (starting with the raw input) into a feature at a higher and more abstract level. Because this process is conducted automatically, it is good at discovering the intricate structures in high-dimensional data without information loss and it requires only very little engineering by human.[12,14,15] Therefore, deep-learning can be quickly applied to tasks with ease and outperform other conventional machine-learning models.

Deep- and machine-learning models are used to obtain the relationship between the predictor and outcome variables, rather than creating a rule based on medical knowledge. Hence, the performance of machine- and deep-learning models is not guaranteed in other situations. Wolpert explains the no-free-lunch theorem; if optimized in one situation, a model cannot produce good results in other situations.[27] Because deep- and machine-learning models can over-fit with the characteristics of hospitals in train data, we conducted a performance test using complete separated test data, which were not used for the model development.

In the present study, we amplified the train dataset from the data of the train group patients by using the methods of collecting multiple datasets from a patient data. Using these methods, we collected a sufficient amount of dataset for developing deep- and machine-learning models. Deep- and machine-learning models require an abundant amount of data for its development. Because the available data are limited, and the outcomes to be predicted are highly diverse in the medical field, this method is promising to future studies in medical domains and will inspire many researchers.

Deep- and other machine-learning predicted models predicted endpoints using different structures as shown in S1 File. The patients for which each model correctly predicted the endpoints also differed. Furthermore, the variable importance of DAHF is different from that of RF, LF, SVM, and BN, as shown in S2 File. Therefore, different algorithms can complement the other weaknesses of the algorithms. We used the combination of two deep-learning algorithm, DNNs, for predicting both in-hospital and long-term mortality simultaneously, and this method was called *ensemble*.[28] Many researchers attempted to improve the accuracy by combining diverse predictive algorithms. This method is also called an *ensemble* algorithm.

Our study has several limitations. First, the deep-learning model is known as a “black box.” Although we can fit and develop the deep-learning based artificial intelligence model, we cannot interpret the model in terms of the approach to the decision of risk score. For example, if the DAHF predicts a high risk of mortality for a patient, the reason for that decision cannot be ascertained. Recently, an interpretable deep-learning model has been studied and will be our next area of study.[29,30] Second, this prediction model was developed with limited variables that could be collected from the KorAHF registry. As shown in previous studies of Cacciatore, functional parameters such as the 6-minute walking test is a good prognostic factor of cardiac diseases.[31] We plan to use more information of HF patients that can enhance the performance of the AI algorithm with more valuable variables. Third, this study was conducted with retrospective big data. Thus, we could not extract accurate past medical histories such as respiratory diseases and malignancies that could also affect long-term mortality. We plan to conduct a prospective study for validating the AI algorithm and confirm the correlation between medical history and HF in our next research.

## Conclusions

In conclusion, we developed and validated a new mortality prediction artificial intelligence model of AHF based on the deep-learning approach. The deep-learning algorithm, DAHF, predicted the in-hospital, 12- and 36-month mortality of patients with AHF more accurately than the existing risk scores and other machine-learning methods. This study showed the feasibility and effectiveness of the deep-learning-based artificial intelligence algorithm model for cardiology, which can be a useful tool for precise decision making in daily practice.

## Supporting information

S1 File Description for development of deep-learning and machine-learning prediction models. (DOCX)

S2 File Difference between each deep-learning and machine-learning prediction model. (DOCX)

## Abbreviations

AHF: acute heart failure
AUC: area under the receiver operating characteristic curve
BN: Bayesian network
DAHF: deep-learning-based artificial intelligence algorithm for predicting mortality of patients with acute heart failure
DNN: deep neural network
GWTG-HF: Get with the Guidelines–Heart Failure
LR: logistic regression
MAGGIC: Meta-Analysis Global Group in Chronic Heart Failure
RF: random forest
SVM: support vector machine

## References

[1] Ponikowski P, Anker SD, AlHabib KF, Cowie MR, Force TL, Hu S, et al Heart failure: preventing disease and death worldwide. ESC Hear Fail. 2014;1: 4–25. 10.1002/ehf2.12005 28834669

[2] Ambrosy AP, Fonarow GC, Butler J, Chioncel O, Greene SJ, Vaduganathan M, et al The global health and economic burden of hospitalizations for heart failure: Lessons learned from hospitalized heart failure registries. J Am Coll Cardiol. Elsevier Inc; 2014;63: 1123–1133. 10.1016/j.jacc.2013.11.053 24491689

[3] Ziaeian B, Fonarow GC. Epidemiology and aetiology of heart failure. Nat Rev Cardiol. 2016;13: 368–378. 10.1038/nrcardio.2016.25 26935038 PMC4868779

[4] Lagu T, Pekow PS, Shieh M-S, Stefan M, Pack QR, Kashef MA, et al Validation and Comparison of Seven Mortality Prediction Models for Hospitalized Patients With Acute Decompensated Heart Failure. Circ Hear Fail. 2016;9. 10.1161/CIRCHEARTFAILURE.115.002912 PMC4988343 27514749

[5] Pocock SJ, Ariti CA, McMurray JJV, Maggioni A, Køber L, Squire IB, et al Predicting survival in heart failure: A risk score based on 39 372 patients from 30 studies. Eur Heart J. 2013;34: 1404–1413. 10.1093/eurheartj/ehs337 23095984

[6] Breiman L. Statistical Modeling: The Two Cultures (with comments and a rejoinder by the author). Stat Sci. 2001;16: 199–231.

[7] Sun G, Shook TL, Kay GL. Inappropriate Use of Bivariable Analysis to Screen Risk Factors for Use in Multivariable Analysis. 1996;49: 907–916. 10.1016/0895-4356(96)00025-x 8699212

[8] Bagley SC, White H, Golomb BA. Logistic regression in the medical literature: standards for use and reporting, with particular attention to one medical domain. J Clin Epidemiol. 2001;54: 979–85. 11576808 10.1016/s0895-4356(01)00372-9

[9] Gulshan V, Peng L, Coram M, Stumpe MC, Wu D, Narayanaswamy A, et al Development and Validation of a Deep Learning Algorithm for Detection of Diabetic Retinopathy in Retinal Fundus Photographs. Jama. 2016;304: 649–656. 10.1001/jama.2016.17216 27898976

[10] Attia ZI, Kapa S, Lopez-Jimenez F, McKie PM, Ladewig DJ, Satam G, et al Screening for cardiac contractile dysfunction using an artificial intelligence–enabled electrocardiogram. Nat Med. 2019;25: 70–74. 10.1038/s41591-018-0240-2 30617318

[11] Kwon J-M, Lee Y, Lee Y, Lee S, Park J. An Algorithm Based on Deep Learning for Predicting In-Hospital Cardiac Arrest. J Am Heart Assoc. 2018;7: e008678 10.1161/JAHA.118.008678 29945914 PMC6064911

[12] LeCun Y, Bengio Y, Hinton G. Deep learning. Nature. 2015;521: 436–444. 10.1038/nature14539 26017442

[13] Lee SE, Lee H-Y, Cho H-J, Choe W-S, Kim H, Choi JO, et al Clinical Characteristics and Outcome of Acute Heart Failure in Korea: Results from the Korean Acute Heart Failure Registry (KorAHF). Korean Circ J. 2017;47: 341 10.4070/kcj.2016.0419 28567084 PMC5449528

[14] Bengio Y, Courville A, Vincent P. Representation Learning: A Review and New Perspectives. IEEE Trans Pattern Anal Mach Intell. 2013;35: 1798–1828. 10.1109/TPAMI.2013.50 23787338

[15] Schmidhuber J. Deep learning in neural networks: An overview. Neural Networks. 2015;61: 85–117. 10.1016/j.neunet.2014.09.003 25462637

[16] Abadi M, Barham P, Chen J, Chen Z, Davis A, Dean J, et al TensorFlow: A System for Large-Scale Machine Learning TensorFlow: A system for large-scale machine learning. 12th USENIX Symp Oper Syst Des Implement (OSDI ‘16).

[17] Kuhn M, Johnson K. Applied Predictive Modeling. New York, NY: Springer New York; 2013.

[18] Mortazavi BJ, Downing NS, Bucholz EM, Dharmarajan K, Manhapra A, Li SX, et al Analysis of Machine Learning Techniques for Heart Failure Readmissions. Circ Cardiovasc Qual Outcomes. 2016;9: 629–640. 10.1161/CIRCOUTCOMES.116.003039 28263938 PMC5459389

[19] Frizzell JD, Liang L, Schulte PJ, Yancy CW, Heidenreich PA, Hernandez AF, et al Prediction of 30-Day All-Cause Readmissions in Patients Hospitalized for Heart Failure. JAMA Cardiol. 2017;2: 204 10.1001/jamacardio.2016.3956 27784047

[20] Fawcett T. An introduction to ROC analysis. Pattern Recognit Lett. 2006;27: 861–874.

[21] Carpenter J, Bithell J. Bootstrap confidence intervals: when, which, what? A practical guide for medical statisticians. Stat Med. 2000;19: 1141–64. 10797513 10.1002/(sici)1097-0258(20000515)19:9<1141::aid-sim479>3.0.co;2-f

[22] Fluss R, Faraggi D, Reiser B. Estimation of the Youden Index and its Associated Cutoff Point. Biometrical J. 2005;47: 458–472. 10.1002/bimj.200410135 16161804

[23] Sartipy U, Dahlström U, Edner M, Lund LH. Predicting survival in heart failure: validation of the MAGGIC heart failure risk score in 51 043 patients from the Swedish Heart Failure Registry. Eur J Heart Fail. 2014;16: 173–179. 10.1111/ejhf.32 24464911

[24] Peterson PN, Rumsfeld JS, Liang L, Albert NM, Hernandez AF, Peterson ED, et al A validated risk score for in-hospital mortality in patients with heart failure from the American heart association get with the guidelines program. Circ Cardiovasc Qual Outcomes. 2010;3: 25–32. 10.1161/CIRCOUTCOMES.109.854877 20123668

[25] Shiraishi Y, Kohsaka S, Abe T, Mizuno A, Goda A, Izumi Y, et al Validation of the Get With The Guideline–Heart Failure risk score in Japanese patients and the potential improvement of its discrimination ability by the inclusion of B-type natriuretic peptide level. Am Heart J. 2016;171: 33–39. 10.1016/j.ahj.2015.10.008 26699598

[26] Khanam SS, Choi E, Son J-W, Lee J-W, Youn YJ, Yoon J, et al Validation of the MAGGIC (Meta-Analysis Global Group in Chronic Heart Failure) heart failure risk score and the effect of adding natriuretic peptide for predicting mortality after discharge in hospitalized patients with heart failure. Garcia de Frutos P, editor. PLoS One. 2018;13: e0206380 10.1371/journal.pone.0206380 30485284 PMC6261415

[27] Wolpert DH. The Supervised Learning No-Free-Lunch Theorems. Proc 6th Online World Conf Soft Comput Ind Appl. 2001; 10–24.

[28] Rokach L. Ensemble-based classifiers. Artif Intell Rev. 2010;33: 1–39.

[29] Chen X, Duan Y, Houthooft R, Schulman J, Sutskever I, Abbeel P. InfoGAN: Interpretable Representation Learning by Information Maximizing Generative Adversarial Nets. 2016.

[30] Fong RC, Vedaldi A. Interpretable Explanations of Black Boxes by Meaningful Perturbation. Proc IEEE Int Conf Comput Vis. 2017;2017–Octob: 3449–3457.

[31] Cacciatore F, Abete P, Mazzella F, Furgi G, Nicolino A, Longobardi G, et al Six-minute walking test but not ejection fraction predicts mortality in ealderly patients undergoing cardiac rehabilitation following coronary artery bypass grafting. Eur J Prev Cardiol. 2012 12;19(6): 1401–9. 10.1177/1741826711422991 21933832

