# Supplementary material for: Artificial intelligence algorithm for predicting mortality of patients with acute heart failure
Source: PLoS One. 2019 Jul 8;14(7):e0219302. doi: 10.1371/journal.pone.0219302 (PMC6613702; doi:10.1371/journal.pone.0219302)
Supplement: S1 File — (DOCX) [file pone.0219302.s001.docx]

**Supplemental File 1. Description for development of deep-learning and machine-learning prediction models**

1. Deep-learning (deep neural network)


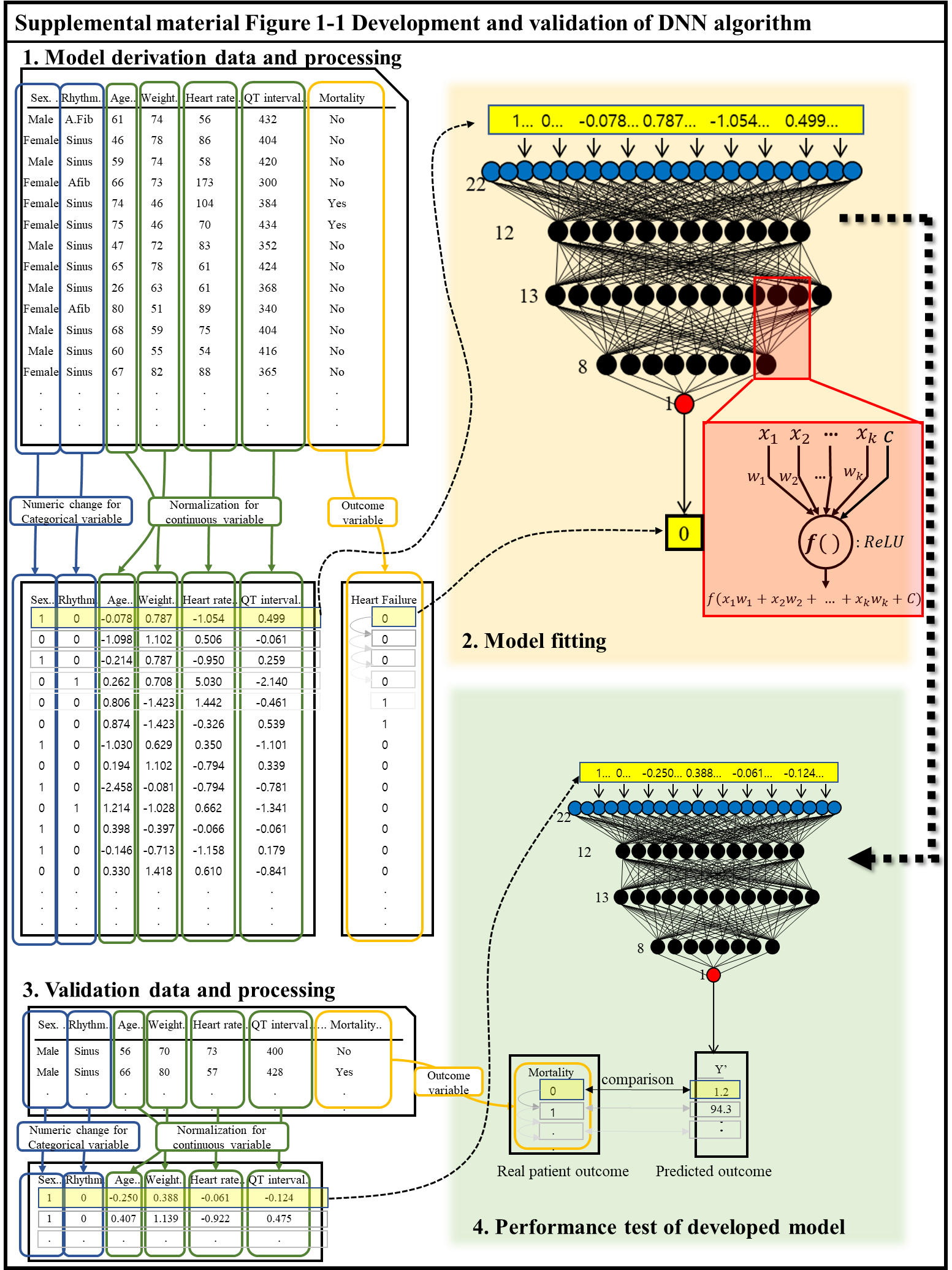


Supplemental material Figure 1-1 shows that deep-learning-based artificial intelligence algorithm for predicting mortality of acute heart failure (DAHF) was developed using a deep neural network (DNN), a deep-learning method.[1–3] Because the accuracy did not increase when more than three hidden layers were added, we used three hidden layers to minimize the parameters to be learned. The input layer consisted of 22 nodes. The first to third hidden layers consisted of 12, 13, and 8 nodes. We used a rectified linear unit (ReLU) as the activation function.[4] The last layer consisted of one node, which represented the risk of mortality and used a sigmoid function. We used batch normalization and dropout layers between hidden layers to decrease overfitting.[5,6]

Before the train dataset for the DAHF development was used, we replaced the values of the categorical variables with binary numeric values (1 or 0) and normalized the values of the continuous variables.[7] This data preprocessing was performed for the train and test datasets separately. To train deep-learning-based model, we input each value of the train data in the input layer and adjusted the weight using the backpropagation method.[7]

We used TensorFlow (the Google Brain Team) as the backend.[8] Furthermore, we used the Adagrad optimizer with default parameters, and binary cross-entropy as the loss function.[9] The value at one node of the DNN is the sum of the values from the upper layer nodes $(x_{1})$ multiplied by their weight ($w_{1})$. The summed value,$(x_{1}w_{1}+x_{1}w_{1}+\cdots+x_{k}w_{k}+C)$, is processed by the activation function, and this value, $ƒ(x_{1}w_{1}+x_{1}w_{1}+\cdots+x_{k}w_{k}+C)$, is sent to the next node.[1–3]

2. Random forest


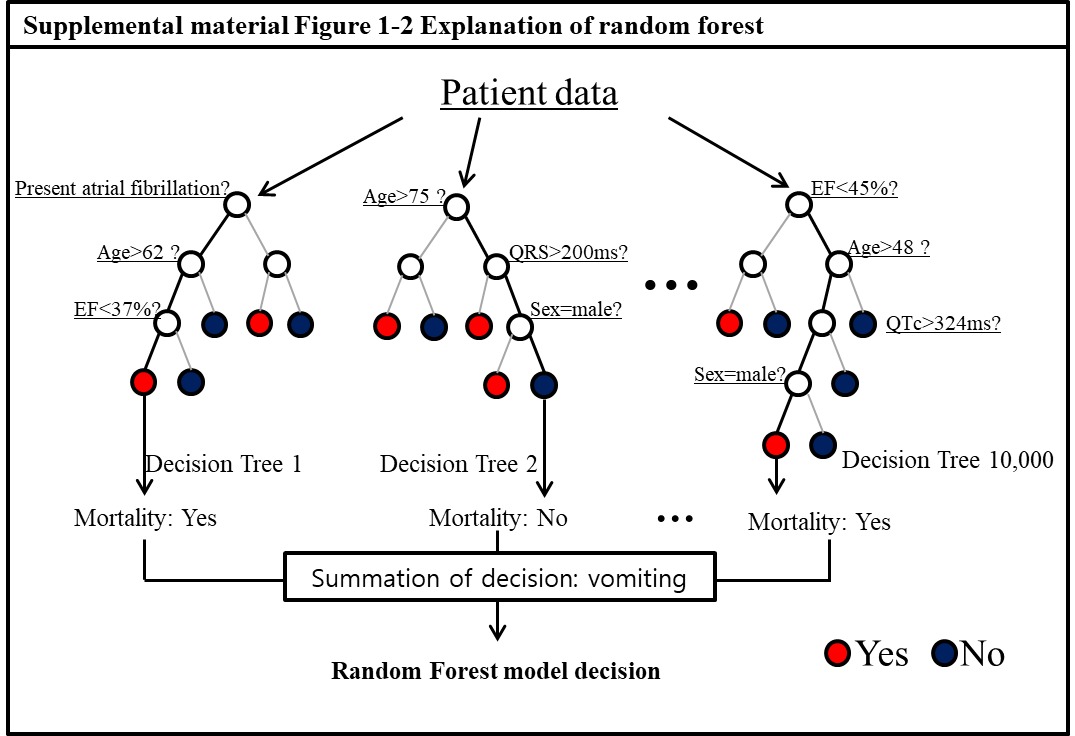


Random forest (RF) is a method that constructs a multitude of decision trees.[10,11] Each decision tree partitions the sample data by splitting the variable at discrete cut-points, as shown in Supplemental material Figure 1-2. Each tree is derived by randomly selecting the dataset from the train dataset, and the RF model concludes with a summary of each decision tree’s prediction. In this study, the RF model consisted of 10,000 decision trees using the *randomForest* package in R (R Development Core Team, Vienna, Austria).[12] The optimal number of the variable, which were randomly sampled as candidates at each split, was determined using 10-fold cross-validation method.

3. Logistic regression

The logistic model is a widely used statistical method. In this model, the logarithm of the odds (log-odds) for the value labeled as “1” is a combination of one or more independent variables. In the present study, the logistic regression imposed stringent constraints on the relationship between explanatory variables and the possibility of mortality. We identified the best logistic regression model among all possible models using the *glmulti* package in R (R Development Core Team, Vienna, Austria).[13] We used original Akaike IC (AIC) as the information criterion and used pairwise interactions. For model selection, we used forward–backward directions.

4. Support vector machine


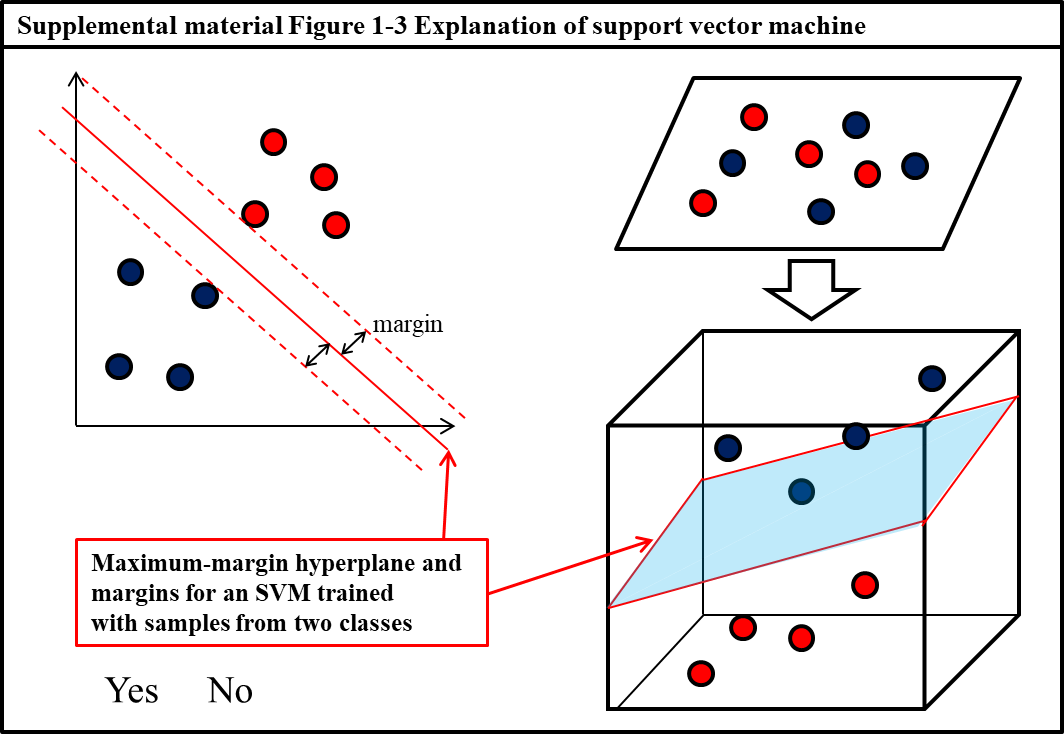


The support vector machine (SVM) utilizes the data points from each outcome class that are closest to the class boundary or misclassified when determining the structure of the boundary.[14] In other words, given the labeled training data, the algorithm outputs an optimal hyperplane to categorize new examples. As shown in Supplemental material Figure 1-3, the optimal separating hyperplane maximizes the margin of the train dataset in the SVM algorithm. If the data are too shuffled to be separated by a straight line, it is necessary to move from a two-dimensional to a three-dimensional view of the dataset, known as kernelling. The SVM model was derived using the *e1071* package in R (R Development Core Team, Vienna, Austria).[15] The radial basis function of the kernel was used for both training and predicting. The optimal value of the cost penalty (3) was determined using 10-fold cross-validation and the parameter needed for the polynomial-type kernel. [14]

5. Bayesian network

The Bayesian network is a probabilistic graphical model that represents a set of variables using Bayesian inference.[16] Their conditional dependencies were represented using a directed acyclic graph. We made a Bayesian network model using *bnlearn* package in R (R Development Core Team, Vienna, Austria).[17] The prediction model consisted with a Bayesian function for generalized linear modeling with independent normal, t, or Cauchy prior distribution for the coefficients. Furthermore, we also calculated 95% credible interval of each variable using the Monte-Carlo estimation method of *MCMC* package in R (R Development Core Team, Vienna, Austria).[18,19]

To make a Bayesian network, we confirmed the correlation networks and plotted a pair-wise correlation graph, connecting the pairs of variables that have a correlation of at least 0.40 as shown in Supplemental material Figure 1-4.

**
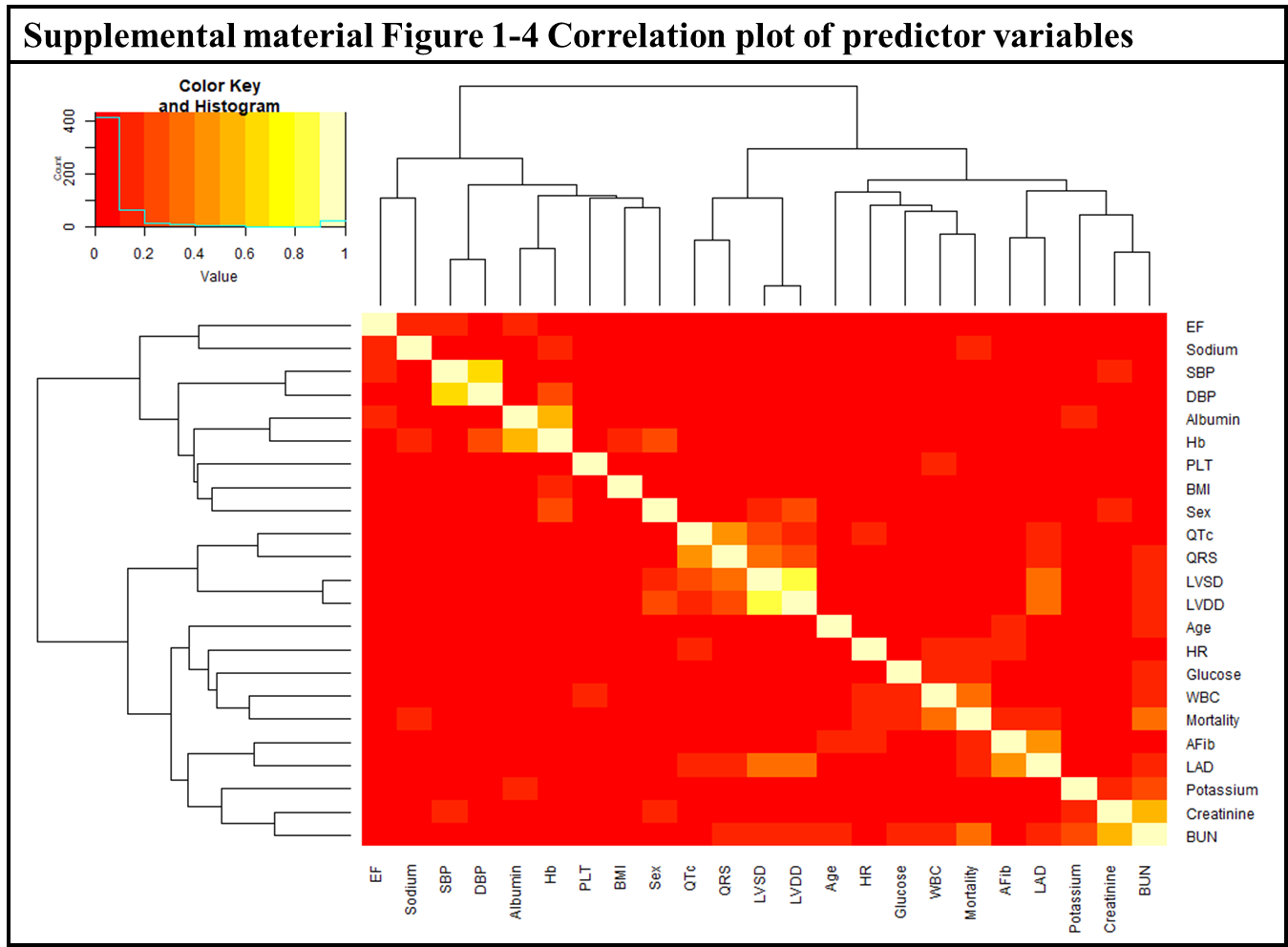
**

Subsequently, we encoded the available prior knowledge in sets of whitelisted and blacklisted arcs to be used in learning the structure of the Bayesian network. Moreover, we produced a consensus Bayesian network model by learning 10,000 Bayesian networks. However, the consensus network was too complex to understand, and we examined the arc strengths above the threshold. Supplemental material Figure 1-5 showed simplified directed acyclic graph (DAG) of Bayesian network prediction model.[20] We confirmed the performance of prediction model by using this Bayesian network.


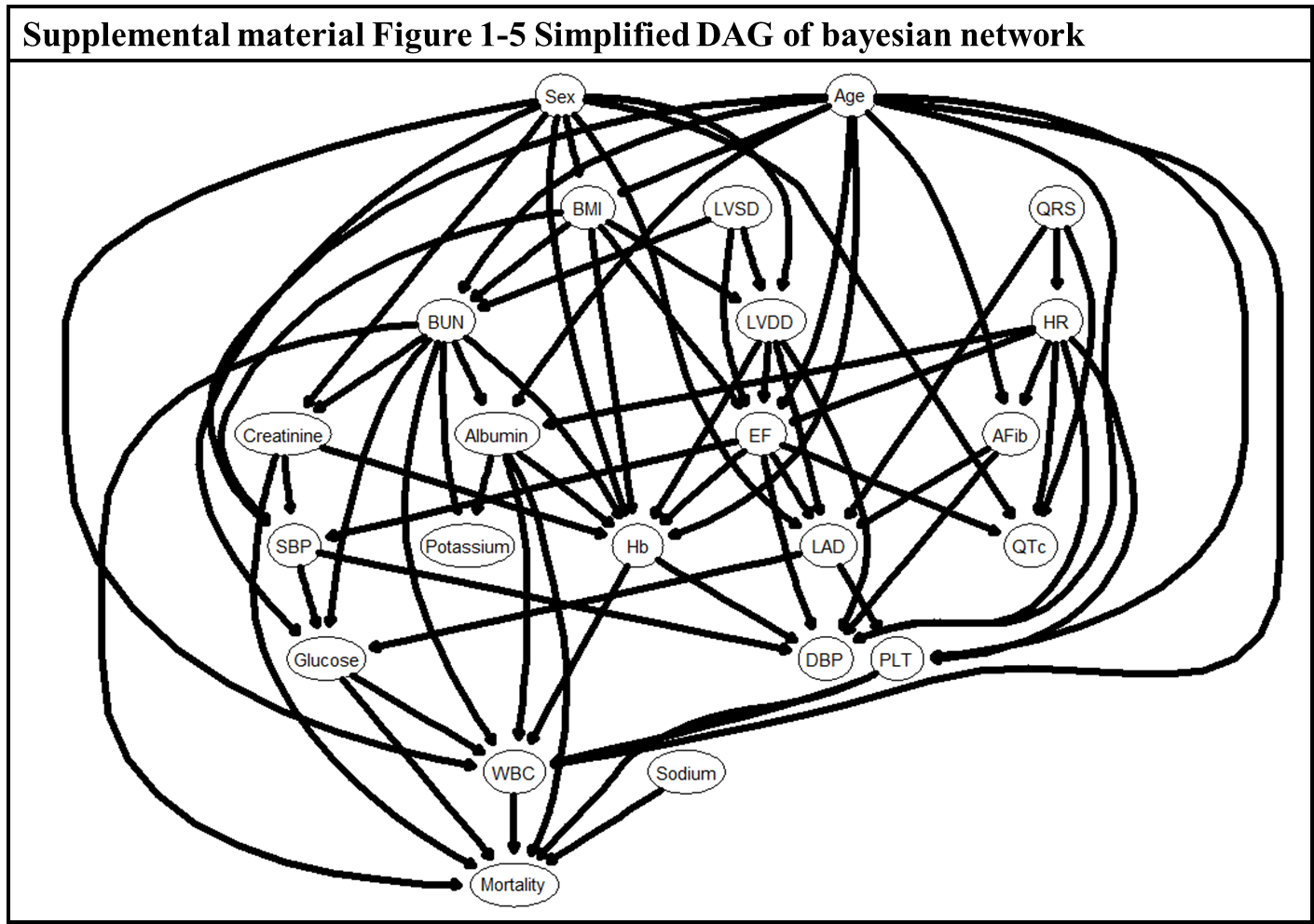


**References**

1. Bengio Y, Courville A, Vincent P. Representation Learning: A Review and New Perspectives. IEEE Trans Pattern Anal Mach Intell. 2013;35: 1798–1828.

2. Schmidhuber J. Deep learning in neural networks: An overview. Neural Networks. 2015;61: 85–117.

3. LeCun Y, Bengio Y, Hinton G. Deep learning. Nature. 2015;521: 436–444.

4. Nair V, Hinton GE. Rectified Linear Units Improve Restricted Boltzmann Machines. Proc 27th Int Conf Mach Learn. 2010; 807–814.

5. Ioffe S, Szegedy C. Batch Normalization: Accelerating Deep Network Training by Reducing Internal Covariate Shift. 2015.

6. Srivastava N, Hinton G, Krizhevsky A, Sutskever I, Salakhutdinov R. Dropout: A Simple Way to Prevent Neural Networks from Overfitting. J Mach Learn Res. 2014;15: 1929–1958.

7. Jayalakshmi T, Santhakumaran A. Statistical Normalization and Backpropagation for Classification. Int J Comput Theory Eng. 2011;3: 89–93.

8. Abadi M, Barham P, Chen J, Chen Z, Davis A, Dean J, et al. TensorFlow: A System for Large-Scale Machine Learning TensorFlow: A system for large-scale machine learning. 12th USENIX Symp Oper Syst Des Implement (OSDI ’16). 2016; 265–284.

9. Kingma DP, Ba J. Adam: A Method for Stochastic Optimization. 2017 IEEE Int Conf Consum Electron ICCE 2017. 2014; 434–435.

10. Kuhn M, Johnson K. Applied Predictive Modeling [Internet]. New York, NY: Springer New York; 2013.

11. Khalilia M, Chakraborty S, Popescu M. Predicting disease risks from highly imbalanced data using random forest. BMC Med Inform Decis Mak. BioMed Central Ltd; 2011;11: 51.

12. Liaw a, Wiener M. Classification and Regression by randomForest. R news. 2002;2: 18–22.

13. Calcagno V, Mazancourt C De. glmulti : An R Package for Easy Automated Model Selection with ( Generalized ) Linear Models. J Stat Softw. 2010;34: 1–29.

14. Chang C-C, Lin C-J. LIBSVM: A library for support vector machines. ACM Trans Intell Syst Technol. 2011;2: 1–27.

15. Karatzoglou A, Meyer D, Hornik K. Support Vector Machines in R. J Stat Softw. 2006;15.

16. Su J, Zhang H. Bayesian network classifiers. Proceedings of the 23rd international conference on Machine learning - ICML ’06. New York, New York, USA: ACM Press; 2006. pp. 897–904.

17. Scutari M. Bayesian Network Constraint-Based Structure Learning Algorithms: Parallel and Optimized Implementations in the bnlearn R Package. J Stat Softw. 2017;77.

18. Hadfield JD. MCMC Methods for Multi-Response Generalized Linear Mixed Models: The MCMCglmm R Package. J Stat Softw. 2010;33: 3050–3076.

19. Smith BJ. boa : An R Package for MCMC Output Convergence Assessment and Posterior Inference. J Stat Softw. 2007;21.

20. Lauritzen SL, Dawid AP, Larsen BN, Leimer H-G. Independence properties of directed markov fields. Networks. 1990;20: 491–505.
