# Supplementary material for: Artificial intelligence algorithm for predicting mortality of patients with acute heart failure
Source: PLoS One. 2019 Jul 8;14(7):e0219302. doi: 10.1371/journal.pone.0219302 (PMC6613702; doi:10.1371/journal.pone.0219302)
Supplement: S2 File — (DOCX) [file pone.0219302.s002.docx]

**Supplemental File 2. Difference between each deep-leaning and machine-learning prediction model**

1. Supplemental material table 2-1. Characteristics of prediction models

|  | **Multivariable logistic regression** | | |  | **Random forest** |  | **Support**  **vector machine** |  | **Bayesian inference** | |  | **Deep neural network** |
| --- | --- | --- | --- | --- | --- | --- | --- | --- | --- | --- | --- | --- |
|  | **HR (95% CI)** | ***p*** | **Dev. diff.** |  | **Mean decrease Gini** |  | **Sens. analysis** |  | **HR (95% credible intervals)** | **Dev. diff.** |  | **AUC diff.** |
| **Demographics** | | | | | | | |  |  |  |  |  |
| Age (years) | 1.034  (1.022–1.047) | <0.001 | 31.4 |  | 57.81 |  | 0.207 |  | 1.036  (1.025–1.049) | 31.3 |  | 0.0117 |
| Male | 2.222  (1.662–2.987) | <0.001 | 29.8 |  | 6.62 |  | 0.000 |  | 2.166  (1.798–2.851) | 29.7 |  | 0.0036 |
| BMI (kg/m^2^) | 0.891  (0.855–0.928) | <0.001 | 32.0 |  | 52.66 |  | 0.236 |  | 0.884  (0.858–0.923) | 31.9 |  | 0.0065 |
| **Vital signs** | | | |  |  |  |  |  |  |  |  |  |
| SBP  (mmHg) | 0.983  (0.973–0.992) | <0.001 | 13.6 |  | 54.11 |  | 0.020 |  | 0.983  (0.974–0.992) | 13.5 |  | 0.0176 |
| DBP  (mmHg) | 1.012 (0.998–1.025) | .082 | 3.0 |  | 34.86 |  | 0.012 |  | 1.012  (0.999–1.028) | 2.9 |  | 0.0097 |
| HR (/min) | 1.014  (1.009–1.019) | <0.001 | 28.3 |  | 17.58 |  | 0.026 |  | 1.014  (1.009–1.018) | 28.2 |  | 0.0063 |
| **Electrocardiography** | | | | | | | | | |  |  | 0.7999 |
| AF (%) | 1.085  (0.796–1.473) | 0.603 | 0.3 |  | 5.27 |  | 0.001 |  | 1.069  (0.775–1.349) | 0.2 |  | 0.0115 |
| QRS duration (ms) | 1.009  (1.003–1.015) | 0.001 | 10.1 |  | 26.62 |  | 0.013 |  | 1.008  (1.003–1.013) | 10.0 |  | 0.0135 |
| QTc (ms) | 0.995  (0.991–0.998) | 0.002 | 10.2 |  | 17.98 |  | 0.078 |  | 0.995  (0.992–0.999) | 10.1 |  | 0.0095 |
| **Echocardiography** | | | | | | | |  |  |  |  |  |
| LAD (mm) | 1.046  (1.034–1.059) | <0.001 | 54.3 |  | 68.48 |  | 0.019 |  | 1.047  (1.035–1.056) | 54.2 |  | 0.0055 |
| LVDd (mm) | 1.042  (1.007–1.077) | 0.017 | 5.7 |  | 42.81 |  | 0.018 |  | 1.046  (1.000–1.076) | 5.6 |  | 0.0106 |
| LVDs (mm) | 0.949  (0.918–0.981) | 0.002 | 9.4 |  | 44.58 |  | 0.013 |  | 0.948  (0.920–0.986) | 9.3 |  | 0.0098 |
| EF (%) | 0.988  (0.975–1.002) | 0.097 | 2.8 |  | 44.55 |  | 0.007 |  | 0.989  (0.973–1.004) | 2.7 |  | 0.0180 |
| **Laboratory test** | | | | | | | |  |  |  |  |  |
| WBC  (/mL) | 1.000  (1.000–1.000) | <0.001 | 258.4 |  | 155.01 |  | 0.109 |  | 1.000  (1.000–1.000) | 258.3 |  | 0.0234 |
| Hb  (g/dL) | 0.859  (0.793–0.930) | <0.001 | 14.2 |  | 68.40 |  | 0.011 |  | 0.869  (0.802–0.925) | 14.1 |  | 0.0067 |
| Platelets  (/mL) | 1.000  (1.000–1.000) | <0.001 | 119.0 |  | 117.10 |  | 0.014 |  | 1.000  (1.000–1.000) | 288.4 |  | 0.0143 |
| Alb  (g/dL) | 0.131  (0.101–0.168) | <0.001 | 288.5 |  | 154.37 |  | 0.019 |  | 0.128  (0.098–0.164) | 118.9 |  | 0.0150 |
| Sodium (mmol/L) | 1.022  (0.997–1.048) | 0.087 | 3.0 |  | 88.96 |  | 0.095 |  | 1.027  (1.002–1.053) | 2.9 |  | 0.0094 |
| Potassium (mmol/L) | 1.464  (1.191–1.802) | <0.001 | 13.1 |  | 43.33 |  | 0.013 |  | 1.496  (1.257–1.806) | 13.0 |  | 0.0035 |
| BUN  (mg/dL) | 1.034  (1.026–1.042) | <0.001 | 69.3 |  | 93.74 |  | 0.015 |  | 1.035  (1.029–1.043) | 69.2 |  | 0.0126 |
| Cr  (mg/dL) | 0.715  (0.619–0.815) | <0.001 | 29.0 |  | 72.78 |  | 0.061 |  | 0.707  (0.614–0.783) | 28.9 |  | 0.0092 |
| Glucose  (mg/dL) | 1.006  (1.004–1.008) | <0.001 | 43.5 |  | 56.78 |  | 0.015 |  | 1.006  (1.004–1.008) | 43.4 |  | 0.0083 |

2. Supplemental material table 2-2. Variable importance of each prediction model

| Rank | **Logistic regression** | **Random Forest** | **Support vector machine** | **Bayesian Network** | **Deep-learning** |
| --- | --- | --- | --- | --- | --- |
| 1 | Alb | WBC | BMI | PLT | WBC |
| 2 | WBC | Alb | Age | WBC | EF |
| 3 | PLT | PLT | WBC | Alb | SBP |
| 4 | BUN | BUN | Sodium | BUN | Alb |
| 5 | LAD | Sodium | QTc | LAD | PLT |
| 6 | Glucose | Cr | Cr | Glucose | QRS |
| 7 | BMI | LAD | HR | BMI | BUN |
| 8 | Age | Hb | SBP | Age | Age |
| 9 | Sex | Age | LAD | Sex | Afib |
| 10 | Cr | Glucose | Alb | Cr | LVDd |
| 11 | HR | SBP | LVDd | HR | LVDs |
| 12 | Hb | BMI | Glucose | Hb | DBP |
| 13 | SBP | LVDs | BUN | SBP | QTc |
| 14 | Potassium | EF | PLT | Potassium | Sodium |
| 15 | QTc | Potassium | LVDs | QTc | Cr |
| 16 | QRS | LVDd | QRS | QRS | Glucose |
| 17 | LVDs | DBP | Potassium | LVDs | Hb |
| 18 | LVDd | QRS | DBP | LVDd | BMI |
| 19 | DBP | QTc | Hb | DBP | HR |
| 20 | Sodium | HR | EF | Sodium | LAD |
| 21 | EF | Sex | Afib | EF | Sex |
| 22 | Afib | Afib | Sex | Afib | Potassium |
